# The efficacy and safety of insulin-sensitizing drugs in HIV-associated lipodystrophy syndrome: a meta-analysis of randomized trials

**DOI:** 10.1186/1471-2334-10-183

**Published:** 2010-06-23

**Authors:** Siddharth H Sheth, Robin J Larson

**Affiliations:** 1 The Dartmouth Institute for Health Policy and Clinical Practice, Lebanon, NH, USA; 2 The VA Outcomes Group, Department of Veterans Affairs Medical Center, White River Junction, VT, USA; 3 Dartmouth Medical School, Hanover, NH, USA

## Abstract

**Background:**

HIV-associated lipodystrophy syndrome (HALS) is characterized by insulin resistance, abnormal lipid metabolism and redistribution of body fat. To date, there has been no quantitative summary of the effects of insulin sensitizing-agents for the treatment of this challenging problem.

**Methods:**

We searched MEDLINE, the Cochrane Library, clinical trial registries, conference proceedings and references for randomized trials evaluating rosiglitazone, pioglitazone or metformin in patients with evidence of HALS (last update December 2009). Two reviewers independently abstracted data and assessed quality using a standard form. We contacted authors for missing data and calculated weighted mean differences (WMD) and 95% confidence intervals (CI) for each outcome.

**Results:**

Sixteen trials involving 920 patients met inclusion criteria. Rosiglitazone modestly improved fasting insulin (WMD -3.67 mU/L; CI -7.03, -0.31) but worsened triglycerides (WMD 32.5 mg/dL; CI 1.93, 63.1), LDL (WMD 11.33 mg/dL; CI 1.85, 20.82) and HDL (WMD -2.91 mg/dL; CI -4.56, -1.26) when compared to placebo or no treatment in seven trials. Conversely, pioglitazone had no impact on fasting insulin, triglycerides or LDL but improved HDL (WMD 7.60 mg/dL; CI 0.20, 15.0) when compared to placebo in two trials. Neither drug favorably impacted measures of fat redistribution. Based on six trials with placebo or no treatment controls, metformin reduced fasting insulin (WMD -8.94 mU/L; CI -13.0, -4.90), triglycerides (WMD -42.87 mg/dL; CI -73.3, -12.5), body mass index (WMD -0.70 kg/m^2^; CI -1.09, -0.31) and waist-to-hip ratio (WMD -0.02; CI -0.03, 0.00). Three trials directly compared metformin to rosiglitazone. While effects on insulin were comparable, lipid levels and measures of fat redistribution all favored metformin. Severe adverse events were uncommon in all 16 trials.

**Conclusion:**

Based on our meta-analysis, rosiglitazone should not be used in HALS. While pioglitazone may be safer, any benefits appear small. Metformin was the only insulin-sensitizer to demonstrate beneficial effects on all three components of HALS.

## Background

While the use of combination antiretroviral therapy (ART) in individuals infected with human immunodeficiency virus (HIV) has led to substantial declines in disease-related morbidity and mortality [1], the benefits have come at a cost. It is estimated that up to 80% of patients receiving ART develop some degree of HIV-associated lipodystrophy syndrome (HALS), characterized by insulin resistance, lipid derangements, and undesirable body fat redistribution [2]. The adverse morphological changes, typified by central fat accumulation and peripheral fat loss, have been associated with threatened confidentiality, poor medication adherence, low self-esteem, and reduced quality of life [3,4]. Further, the metabolic changes associated with HALS may increase the risk of cardiovascular disease [5-9], a consequence of growing relevance as the life expectancy of people with HIV continues to improve [10].

A common initial approach to slowing or reversing the undesirable changes associated with HALS has been to adjust antiretroviral regimens by eliminating thymidine analogues [11-13]. While this approach has been shown to slow progression, it appears to have less impact on reversing existing disease. Further, recent reviews have concluded that the effects of making such ART switches are generally modest and slow to take effect [11,14,15]. As a result, focus has shifted to evaluating interventions targeted at specific components of HALS.

Interest in the biguanide metformin and the thiazolidinediones rosiglitazone and pioglitazone has stemmed in part from the documented efficacy of all three drugs for improving insulin sensitivity [16,17] and reducing the progression from impaired glucose tolerance to diabetes [18-20] in non-HIV populations. In addition, some have theorized that these drugs might have a special role in addressing HALS. Metformin has been shown to promote weight maintenance or loss [20] rather than the weight gain seen with most hypoglycemic agents and was recently found to increase HDL3-cholesterol and reduce immature forms of HDL in patients with HALS [21]. Similarly, thiazolidinediones have shown promise because of their agonist action at peroxisome proliferator-activated-γ (PPARγ) receptors [22]. PPARγ is known to exhibit preferential expression in subcutaneous adipose tissue and has been associated with genetic forms of lipodystrophy where PPARγ genes were absent [23]. Further, studies have found that PPARγ agonists improve insulin sensitivity and increase subcutaneous adipose tissue mass in HIV negative populations with lipoatrophic diabetes [24].

While several randomized controlled trials have evaluated insulin-sensitizing agents for the treatment of HALS, results have been mixed and prior efforts to pool the data have been limited to qualitative summarizations [25,26]. In addition, recent data have raised safety concerns about the use of thiazolidinediones in non-HIV populations[27]. In this paper, we present the first quantitative meta-analysis of randomized-controlled trials evaluating the effects of rosiglitazone, pioglitazone or metformin on insulin, glucose, lipids, body fat redistribution and adverse events in patients with evidence of HALS.

## Methods

### Search Strategy

We searched MEDLINE (1996 to December 2009) using the following key words: HIV/AIDS, lipodystrophy, insulin sensitivity, thiazolidinediones, rosiglitazone, pioglitazone, biguanides and metformin; additionally we exploded medical subject headings related to these key words. These results were combined with Phases 1 and 2 of a highly sensitive search strategy recommended for identifying all randomized trials in Medline [28]. No language restrictions were employed. Using similar terms, we also searched the Cochrane Central Register of Controlled Trials, the Cochrane Database of Systematic Reviews and the Database of Abstracts of Reviews of Effects (all from The Cochrane Library, Issue 4, 2009). In order to locate published articles not identified by our electronic searches, we manually reviewed references of studies that met our inclusion criteria as well as any other articles or reviews that were relevant to our study question. To identify unpublished studies, we electronically searched clinical trial registries including ClinicalTrials.gov (December 2009), and manually searched abstracts from scientific conference proceedings including the Infectious Diseases Society of America Annual Meeting (1997-2009) and the Conference on Retroviruses and Opportunistic Infections (1997-2009).

### Study Selection Criteria

Two reviewers independently evaluated studies for eligibility using the following inclusion criteria: 1) the design was a randomized controlled trial (RCT); 2) the intervention was either rosiglitazone, pioglitazone or metformin, 3) the comparison was either rosiglitazone, pioglitazone, metformin, placebo or no treatment; 4) the population consisted of subjects with one or more features of HIV-associated lipodystrophy (e.g., body fat redistribution, insulin resistance, hypertriglyceridemia); and 5) the subjects were followed for a minimum duration of 4 weeks. We did not employ any exclusion criteria.

### Data Abstraction

Two reviewers independently abstracted data from included studies using a standardized form. Discrepancies were resolved through discussion and review of the source document. We contacted authors if essential data was not provided in the published reports.

### Quality Assessment

Two reviewers independently assessed each study's methodological quality using the Jadad Scale [29], which assesses randomization, double-blinding, and attrition based on seven questions. Scores range from one to five points with higher quality studies receiving a score greater than or equal to three. We resolved differences in quality assessment through joint discussion and review of the source document.

### Outcomes measures

#### Primary outcomes-fasting insulin and glucose levels

We designated fasting insulin and fasting glucose as our primary outcomes of interest for several reasons--they were the most commonly reported measures, have high clinical familiarity, and are both established clinical markers for diabetes and cardiovascular mortality [30]. While we had also intended to evaluate one or more formal measures insulin sensitivity, the wide variation in methodology and reporting across trials precluded quantitative summarization.

#### Secondary outcomes-fasting lipid levels, body fat distribution, adverse events

We designated fasting lipid levels, body fat distribution, and adverse events as secondary outcomes. To assess the impact of the insulin-sensitizers on fasting lipid levels we selected the clinically relevant components--HDL-cholesterol, LDL-cholesterol, and triglycerides. Since the most meaningful measures of body fat redistribution in HALS are not known, methods for assessing the morphologic changes varied widely across trials. We selected body mass index (BMI) and waist to hip ratio (WHR), because they were widely reported, are clinically familiar, and have known associations with cardiovascular risk [31,32]. We also assessed visceral abdominal fat, because it was the most commonly reported direct measure of central fat deposition. Unfortunately, methods for directly measuring peripheral fat wasting were too varied to permit quantitative summarization. Lastly, in order to capture clinically important adverse events, we chose the most consistently defined and reported measure--severe adverse events, defined as Grade 3 or 4 or resulting treatment discontinuation or study withdrawal.

### Data Synthesis

#### Pooled Summary Analysis

For each outcome of interest, we grouped the reported findings according to the interventions being compared: (1) rosiglitazone versus placebo or no treatment (2) pioglitazone versus placebo (3) metformin versus placebo or no treatment, or (4) rosiglitazone versus metformin. All efficacy outcomes were reported as continuous variables. We calculated summary weighted mean differences based on the means and standard deviations of the change from baseline for each study arm using random-effects models. When this data was not provided in the manuscript, we requested missing information from authors. If requests were declined or not responded to after at least two inquiries, we attempted to calculate or estimate the values from data that was available using established methods [33] (Additional File 1). Appropriate conversion factors were used to convert the data to uniform units. For one study [34], data for fasting insulin and fasting glucose had to be extrapolated from figures. For another study [35] that provided all of the needed data for fasting insulin except the standard deviation around the change from baseline for the placebo group, we used a conservative estimate based on the standard deviations found in other trials. All calculations were performed using RevMan 4.2.6.

#### Heterogeneity and Publication Bias

We formally assessed for heterogeneity amongst the trials contributing to each summary estimate using the chi-square test and considered a p-value of less than 0.10 to indicate significant heterogeneity [33]. For each summary estimate in which heterogeneity was present, we reviewed the results of the contributing studies in effort to identify the source of variability. If one or more outliers could be identified, we performed sensitivity analyses to investigate how their removal impacted the summary estimate.

In order to assess for publication bias, we constructed funnel plots based on our primary outcomes of interest, fasting insulin and fasting glucose. We visually inspected the plots for evidence of a paucity of small studies with negative or less robust results that might suggest publication bias. All heterogeneity and publication bias assessments were performed using Revman 4.2.6.

## Results

### Identification of Studies

Of the 56 potentially relevant studies identified by our search strategy, we excluded 30 studies because they were not RCTs or did not report any of our outcomes of interest (Additional File 2). After full text review of the remaining 26 studies, we excluded an additional five studies because their population, comparison, or outcome of interest did not meet our inclusion criteria. Further, we excluded one unpublished abstract (el Bejjani) for which we were unable to obtain adequate details. This left 16 unique RCTs, represented by 20 published manuscripts [34-53] for inclusion in the meta-analysis.

### Study Characteristics

Additional File 3 presents the characteristics of the 16 trials that met our inclusion criteria. Studies were based in North America, Europe, and Australia and sample sizes ranged from 13 to 130. In total, 920 HIV-infected subjects were studied, consisting predominantly of men (56-100%) in their forties (mean ages 40-48 years) with mean CD4 counts between 340 and 637 cells/mm^3 ^and varying HIV lipodystrophy criteria and ART regimens. Study durations ranged from 8 to 48 weeks and the overall attrition rates were generally very low. For the three studies that exceeded 10% attrition [35,37,43], the rates of drop-out were well balanced between study arms. Fifteen of the 16 trials received a Jadad score greater than or equal to 3, the threshold indicating higher methodological quality.

### Rosiglitazone versus Placebo or No Treatment

**Figure 1:**
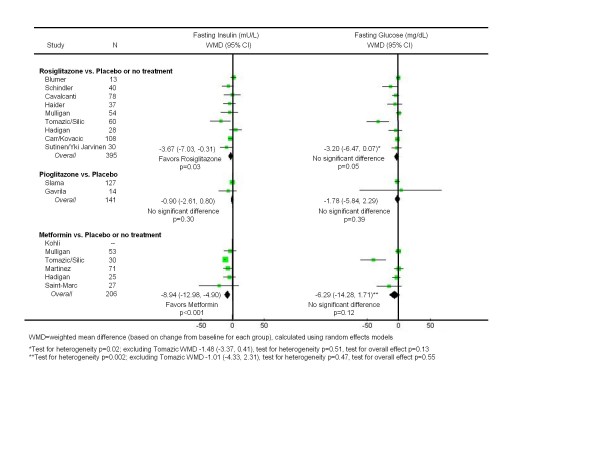
**Effects of insulin sensitizing drugs versus placebo or no treatment on fasting insulin and glucose levels in patients with HIV-associated lipodystrophy syndrome**.

**Figure 2:**
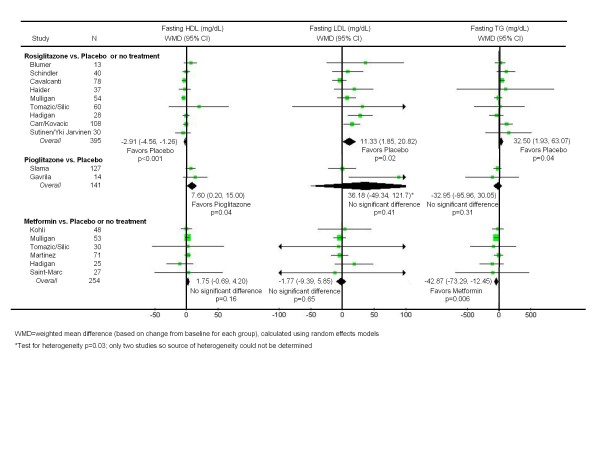
**Effects of insulin sensitizing drugs versus placebo or no treatment on fasting lipids levels in patients with HIV-associated lipodystrophy syndrome**.

**Figure 3:**
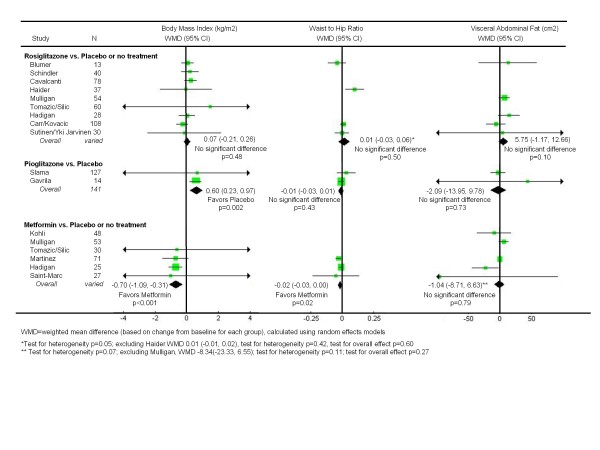
**Effects of insulin sensitizing drugs versus placebo or no treatment on body weight and morphology in patients with HIV-associated lipodystrophy syndrome**.

Nine unique trials compared rosiglitazone to placebo or no treatment in 470 subjects over a mean duration of 26 weeks [35-37,41,42,44,47-50,52,53]. As shown in Figure 1, rosiglitazone resulted in a modest decrease in fasting insulin (WMD -3.67 mU/L; CI -7.03, -0.31, p = 0.03), but had no significant effect on fasting glucose. On the other hand, rosiglitazone had clearly unfavorable effects on lipid levels (Figure 2). Compared to placebo or no treatment, it significantly increased LDL-cholesterol (WMD 11.3 mg/dL; CI 1.85, 20.8, p = 0.02) and triglycerides (WMD 32.5; CI 1.93, 63.1), and significantly worsened HDL-cholesterol (WMD -2.91 mg/dL; CI -4.56, -1.26, p < 0.001). As shown in Figure 3, rosiglitazone had no significant effect on any of the body fat outcomes.

### Pioglitazone versus Placebo

Two trials evaluated pioglitazone to placebo in 144 subjects over a mean duration of 50 weeks [39,51]. As shown in Figure 1, pioglitazone had no impact on fasting insulin or glucose levels. However, in contrast to the negative lipid effects of rosiglitazone, pioglitazone significantly improved HDL-cholesterol (WMD 7.60 mg/dL; CI 0.20, 15.0, p = 0.04) (Figure 2). The findings for LDL-cholesterol were heterogeneous, with the larger trial [51] reporting no significant difference between the study arms. As shown in Figure 3, pioglitazone had no significant effect on waist-to-hip ratio or visceral adipose tissue. Compared to placebo, pioglitazone increased the mean body mass index (WMD 0.60 kg/m2; CI 0.23, 0.97, p = 0.002).

### Metformin versus Placebo or No Treatment

Six unique trials compared metformin to placebo or no treatment in 287 subjects over a mean duration of 27 weeks [35,40,43,45-47,49]. As shown in Figure 1, metformin led to a significant decrease in fasting insulin (WMD -8.94 mU/L; CI -13.0, -4.90, p < 0.001). With regard to lipid profiles (Figure 2), metformin had no significant impact on HDL or LDL-cholesterol, but significantly lowered triglyceride levels (WMD -42.87 mg/dL; CI -73.3, -12.5, p = 0.006). Unlike the thiazolidinediones, metformin also led to significant reductions in BMI (WMD -0.70 kg/m^2^; CI -1.09, -0.31, p < 0.001) and waist-to-hip ratios (WMD -0.02; CI -0.03, 0.00, p = 0.02) (Figure 3). While the findings for visceral abdominal fat were not significant, the point estimate suggested a slight improvement, which became larger when the heterogeneous study [35] was removed.

### Rosiglitazone versus Metformin

**Figure 4:**
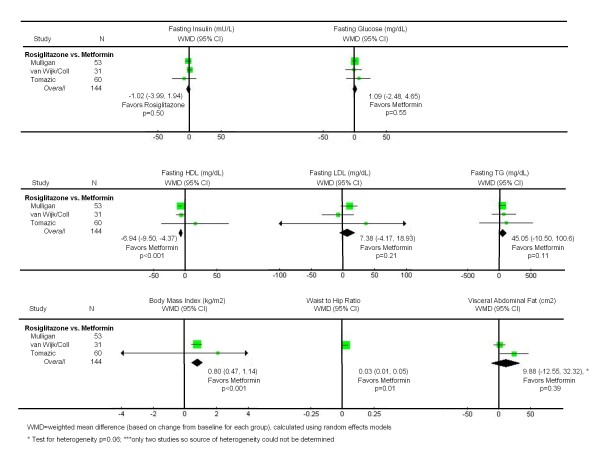
**Effects of rosiglitazone vs. metformin on insulin sensitivity, lipid profiles, and morphology in patients with HIV-associated lipodystrophy syndrome**.

Three unique trials [34,35,38,47,49] compared rosiglitazone and metformin head-to-head in 152 subjects over a mean duration of 29 weeks (Figure 4). There were no statistically significant differences between the two drugs with regard to fasting insulin or glucose levels. All three lipid findings were less favorable for rosiglitazone when compared to metformin, including significant reductions in HDL-cholesterol (WMD -6.94 mg/dL; CI -9.50, -4.37, p < 0.001). Relative changes in body mass index and waist-to-hip ratio were also statistically significantly less favorable with rosiglitazone (WMD 0.80 kg/m^2^; CI 0.47, 1.14, p < 0.001) and (WMD 0.03; CI 0.01, 0.05, p = 0.01), respectively.

### Severe Adverse Effects

Severe adverse events were defined as Grade 3 or 4 or leading to treatment discontinuation or study withdrawal (Additional File 4). Six studies [39,42,46,48,52,53] did not explicitly report severe adverse event outcomes and four [34,40,45,49] reported no events in either arm. Based on the remaining six trials [35-37,39,41,51] in which at least one severe adverse event was reported, the events were generally uncommon and varied widely in nature. Expected side effects (anemia and gastrointestinal (GI) problems with rosiglitazone; elevated ALT levels and weight gain with pioglitazone; and GI problems metformin) were observed in both intervention and control study arms. Changes in lactate were not statistically different between study arms in the few studies that report this outcome.

### Heterogeneity and Publication bias

Five of the 31 summary estimates did not pass their respective tests for heterogeneity, indicating that the findings of the contributing studies varied beyond what would be expected by chance alone. Nevertheless, in four cases we were able to identify a single outlying study that was responsible; in the fifth case there were only two studies being combined (Figures 1, 2, 3. In no case did removing the outlying study result in a change in the direction or statistical significance of the original summary estimate. We found no evidence of publication bias based on visual inspection of funnel plots for the primary outcome measures fasting insulin and fasting glucose.

## Discussion

The findings of our meta-analysis of randomized trials evaluating insulin-sensitizing drugs in HALS differed according to the drug studied. We found that rosiglitazone modestly improved fasting insulin, but worsened fasting lipid levels and had no favorable impact on measures of central adiposity. Conversely while pioglitazone had no effect on fasting insulin or glucose, it improved fasting HDL-cholesterol without negative effects on other lipids. It too had no favorable impact on measures of central adiposity. In contrast, metformin favorably impacted outcomes across all three areas of interest including statistically significant reductions in fasting insulin, fasting triglycerides, body mass index and waist-to-hip ratio. Head-to head trials reinforced the findings of placebo- and no treatment-controlled trials with metformin demonstrating more favorable impacts on lipids and body fat redistribution, compared to rosiglitazone. Severe side effects were uncommon with all three drugs.

### Overall completeness and applicability of evidence

Though sixteen trials involving over 920 subjects met our inclusion criteria, the completeness of the data differed according to intervention. While rosiglitazone and metformin were both evaluated in multiple trials, the findings for pioglitazone were based on only two trials, one of which had just 14 subjects. With regard to generalizability, our inclusion criteria allowed for a wide range of HALS populations, which varied by both the character and severity of their lipodystrophy manifestations and the composition of their ART regimens. Subjects were predominantly male but females comprised 25-50% of several trials. The trial settings and drug dosing were consistent with those found in clinical practice. Important groups that were not represented in the study populations include adolescents and children with HALS.

### Quality of the evidence

In assessing the methodological quality of the included studies, we found that two trials used "no treatment" groups rather than placebo controls [46,47,49] and thus could have been susceptible to performance bias. Both of these unblinded trials favored the intervention group more strongly than comparable blinded trials for the fasting insulin and fasting glucose outcomes, thereby possibly inflating these summary estimates. Additionally, several trials did not adequately describe their method of randomization or allocation concealment thus precluding formal assessment of the likelihood of selection bias.

### Potential biases in the review process

It is possible that we missed relevant trials, although we believe this is unlikely based on our systematic search efforts and no evidence of publication bias. We are aware of several ongoing trials [54-56] evaluating pioglitazone which, when available, should allow for a more robust analysis of the treatment's efficacy and safety.

In regard to our inclusion criteria, it is possible that allowing populations with diverse manifestations of HALS and continued use of certain antiretroviral agents may have contributed to the failure of several studies to find statistically significant differences for particular clinical outcomes. For example, subjects included based on isolated derangements in one element of HALS (i.e. central adiposity) might have been normal or only mildly affected with regard to other elements (i.e. insulin sensitivity) and thus would have had little if any room to benefit with respect to the latter measure. Similarly, because some antiretroviral agents including specific NRTIs and PIs, have been shown to increase central adiposity [57] and down-regulate PPAR receptors [35-37], their continued use could have diluted or negated potential beneficial effects of the interventions. Nonetheless, despite variation in ART usage across trials, nearly all summary estimates passed their tests for heterogeneity.

In regards to our analysis, our summary estimates for the body morphology outcomes may be less robust or generalizable than other outcomes due to reporting in only a small subset of studies. Furthermore, while the most consistently reported morphology-related variables--BMI, WHR and VAT--capture elements of central adiposity, they do not specifically address peripheral wasting, which may be particularly important in the decision to use metformin in patients with lipoatrophy [43,58].

### Agreements and disagreements with other studies or reviews

This is the first quantitative meta-analysis evaluating the effect of insulin-sensitizers in HALS. In 2004 Benavides [25] sought to summarize the efficacy and safety any pharmacological therapy for the treatment of HALS, in particular the effects on fat redistribution. While the review concluded that "no drug exists to fully ameliorate or correct the cosmetic changes of HALS", the review was limited by studies with non-randomized designs and the inability to quantitatively assess the effects of specific agents. In 2007 McGoldrick [26] reviewed randomized trials of statins, fibrates, and insulin-sensitizers for managing dyslipidemias in HIV-infected subjects taking ART. While concluding that "most studies suggested beneficial effects and satisfactory safety profiles", the review also warned that "rosiglitazone appeared to have some detrimental effects on lipid profiles". Due to limitations of data reporting, again no quantitative summarizations were performed.

When placed in the context of studies evaluating insulin-sensitizers in non-HIV populations, our findings are consistent with growing safety concerns surrounding the use of thiazolidinediones, most notably rosiglitazone, due to increased cardiovascular events[27]. The American Diabetes Association and the European Association for the Study of Diabetes now specifically recommend against the use of rosiglitazone, and while pioglitazone is still included in the management pathway, is considered "less validated"[59]. Additionally our positive findings for metformin are consistent with a recent review of the medication in persons at risk for diabetes mellitus which showed favorable impacts on lipid profiles, insulin resistance, and BMI over a mean study period of 1.8 years in 31 study trials[60].

### Implications for research

Ongoing efforts to identify and compare potential interventions for HALS would benefit from several actions. First, standardization of the methods used to evaluate insulin sensitivity and body morphological changes, including measures of both central adiposity and peripheral wasting, would greatly facilitate comparisons across studies and interventions. In addition, the inclusion of additional measures such as quality of life and ART compliance could capture important effects not revealed by changes in laboratory and imaging studies alone. Similarly, longer term studies of the more promising interventions are needed to fully assess whether the short-term changes in surrogate markers of cardiovascular risk translate into long-term benefits in reduced cardiovascular events and mortality.

### Implications for practice

The findings of our meta-analysis suggest several implications for practice. With regard to rosiglitazone, we believe there is adequate evidence suggesting that the drug should not be given to patients with HALS. While pioglitazone did not appear to cause the adverse lipid problems seen with rosiglitazone, the number of completed clinical studies is small and more data is needed to determine whether it is an effective treatment option for patients with HALS. Lastly, while the evidence showed that metformin had favorable effects across all three components of HALS, whether these short-term, surrogate changes will translate to long-term clinical benefits is not known.

## Competing interests

The authors declare that they have no competing interests.

## Authors' contributions

SS conceived the study, participated in the design and coordination of the study, drafted and edited the manuscript, and has given final approval for the manuscript to be published.

RL participated in the design and coordination of the study, drafted and edited the manuscript, and has given final approval for the manuscript to be published.

## Pre-publication history

The pre-publication history for this paper can be accessed here:

http://www.biomedcentral.com/1471-2334/10/183/prepub

## Supplementary Material

Additional file 1 **Methods - Pooled Summary Analysis**. A detailed description of how the pooled summary analysis was discussed in this section. Click here for file

Additional file 2 **Figure - Identification of Studies**. This figure displays the process for identifying the 20 RCT's that were included in the meta-analysis. Click here for file

Additional file 3 **Table S1. Baseline Characteristics**. Baseline characteristics of randomized controlled trials included in the meta analysis. Click here for file

Additional file 4 **Table - Severe Adverse Effects**. This table summarizes the severe effects that were found in each study quantitatively. Additionally, specific types of severe effects were quantified by intervention. Click here for file
