# Supplementary material for: The efficacy and safety of insulin-sensitizing drugs in HIV-associated lipodystrophy syndrome: a meta-analysis of randomized trials
Source: BMC Infect Dis. 2010 Jun 23;10:183. doi: 10.1186/1471-2334-10-183 (PMC2906460; doi:10.1186/1471-2334-10-183)
Supplement: Additional file 1 — Methods - Pooled Summary Analysis. A detailed description of how the pooled summary analysis was discussed in this section. [file 1471-2334-10-183-S1.DOC]

**Appendix**

Methods

*Pooled Summary Analysis*

In cases in which studies provided data for baseline (b) and final (f) time points, we calculated the mean (*X*) of the change (c) as, *X*c=*X*f – *X*b and the standard deviation (SD) of the change as, SDc=$\sqrt{(SDb^{2}+SDf^{2}-(2\times p\times SDb\times SDf)}$ where p is the correlation between the baseline and final measurements. While the value of p can range from 0 indicating no correlation to 1 indicating a perfect correlation, for most clinical outcomes

it is expected that there would be at least a modest correlation between the baseline and

final values. In addition, while it has been shown that varying the value of p can have negligible impact on the findings of the meta-analysis , setting p at 0.50 has been recommend[56]. We chose a slightly more conservative value of 0.40 as was used in a recent meta-analysis evaluating changes in lipid levels with garlic [57]. When standard error (SE) values were provided rather than SD, we converted them directly (SD=$SE\times\sqrt{N}$). If studies provided only 95% confidence intervals around the reported mean, we calculated the SD as,$\sqrt{N\times(\text{upper limit of CI-lower limit of CI)/t-value}^{2}}$.

Results – Study Flow Chart (Figure) and Severe Adverse Events (Table)

*Please see attached figure and table.*
