# Supplementary material for: The efficacy and safety of insulin-sensitizing drugs in HIV-associated lipodystrophy syndrome: a meta-analysis of randomized trials
Source: BMC Infect Dis. 2010 Jun 23;10:183. doi: 10.1186/1471-2334-10-183 (PMC2906460; doi:10.1186/1471-2334-10-183)
Supplement: Additional file 2 — Figure - Identification of Studies. This figure displays the process for identifying the 20 RCT's that were included in the meta-analysis. [file 1471-2334-10-183-S2.PPT]

## Slide 1
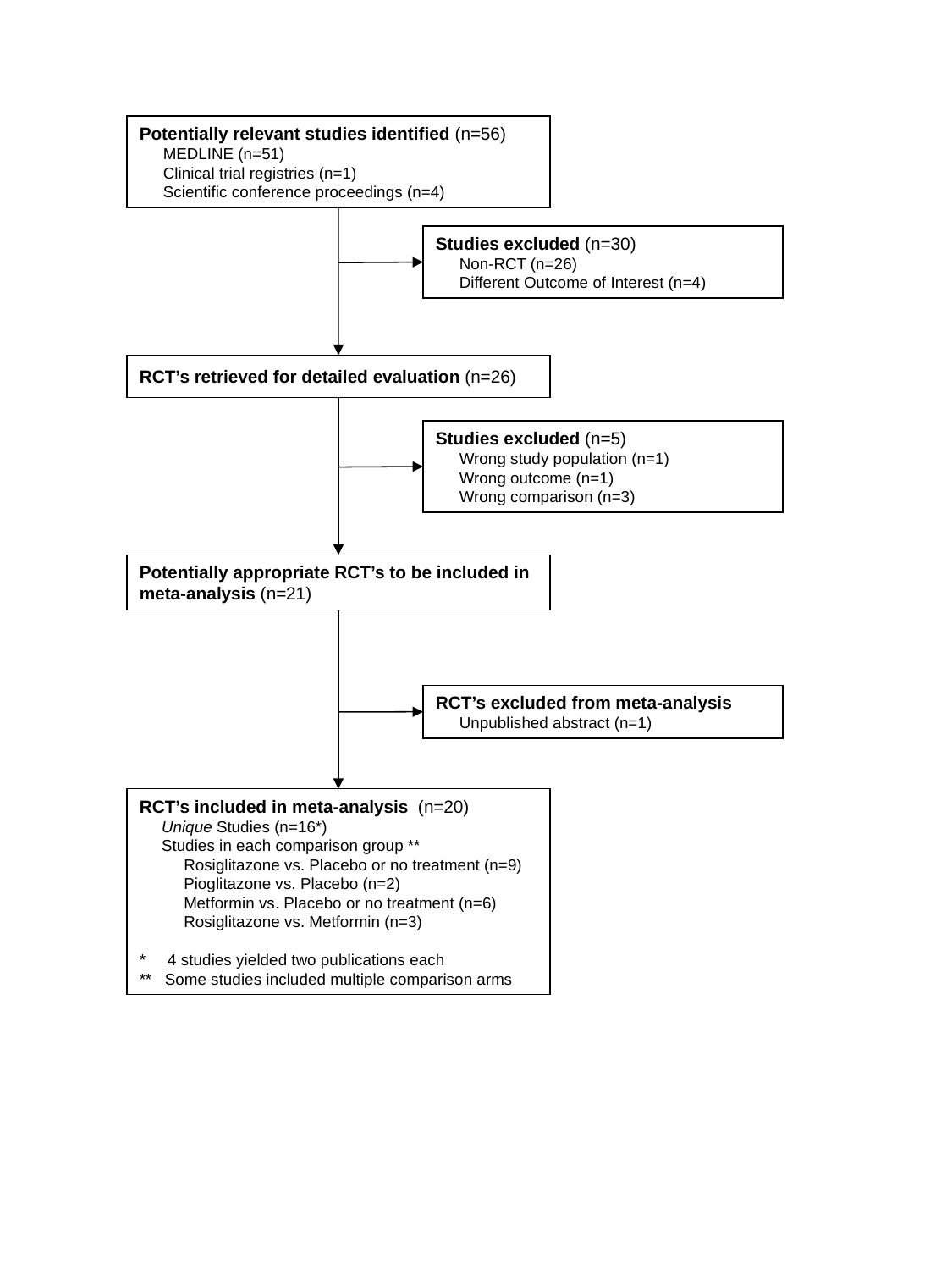

Potentially relevant studies identified (n=56)
MEDLINE (n=51)
Clinical trial registries (n=1)
Scientific conference proceedings (n=4)
Studies excluded (n=30)
 	Non-RCT (n=26)
	Different Outcome of Interest (n=4)
RCT’s retrieved for detailed evaluation (n=26)
Studies excluded (n=5)
Wrong study population (n=1)
Wrong outcome (n=1)
Wrong comparison (n=3)
Potentially appropriate RCT’s to be included in meta-analysis (n=21)
RCT’s excluded from meta-analysis
Unpublished abstract (n=1)
RCT’s included in meta-analysis (n=20)
 Unique Studies (n=16*)
 Studies in each comparison group **
 Rosiglitazone vs. Placebo or no treatment (n=9)
 Pioglitazone vs. Placebo (n=2)
 Metformin vs. Placebo or no treatment (n=6)
 Rosiglitazone vs. Metformin (n=3)
* 4 studies yielded two publications each
** Some studies included multiple comparison arms
