# Supplementary material for: The efficacy and safety of insulin-sensitizing drugs in HIV-associated lipodystrophy syndrome: a meta-analysis of randomized trials
Source: BMC Infect Dis. 2010 Jun 23;10:183. doi: 10.1186/1471-2334-10-183 (PMC2906460; doi:10.1186/1471-2334-10-183)
Supplement: Additional file 3 — Table S1. Baseline Characteristics. Baseline characteristics of randomized controlled trials included in the meta analysis. [file 1471-2334-10-183-S3.DOC]

**Table 1**. Baseline characteristics of randomized controlled trials evaluating insulin sensitizing drugs for HIV-associated lipodystrophy syndrome

| Reference | Country | Number | Mean Age (years) | | | Gender (% male) | | Mean CD4 (cells/mm3) | | Required element(s) of Lipodystrophy | | Current NRTI* use (%) | | Current PI** use (%) | | Intervention (dose) | | Comparison (dose) | | Duration of Follow Up | | Completeness of Follow Up (%) | | Jadad Score**** |
| --- | --- | --- | --- | --- | --- | --- | --- | --- | --- | --- | --- | --- | --- | --- | --- | --- | --- | --- | --- | --- | --- | --- | --- | --- |
| **Rosiglitazone versus Placebo or no treatment** | | | | | |  | |  | |  | |  | |  | |  | |  | |  | |  | |  |
| Blumer (2009) | The Netherlands | 13 | 44 | | | 100 | | 555 | | lipoatrophy | | NR | | 0 | | rosiglitazone (8mg daily) | | Placebo | | 16 weeks | | 100 | | 5 |
| Schindler (2009) | Austria | 44 | 46 | | | 93 | | 527 | | morphologic plus insulin resistance or dyslipidemia | | 73 | | 25 | | rosiglitazone (4mg daily) | | Placebo | | 6 months | | 91 | | 3 |
| Cavalcanti (2007) | Canada | 96 | 47 | | | 97 | | 442 | | morphologic | | 99 | | 100 | | rosiglitazone (4mg daily) | | Placebo | | 24 weeks | | 81 | | 5 |
| Mulligan*** (2007) | USA | 54 | 45 | | | 65 | | 619 | | morphologic; insulin resistance | | 93 | | 67 | | rosiglitazone (4mg daily) | | Placebo | | 16 weeks | | 91 | | 4 |
| Haider (2007) | Austria | 37 | 19-50 | | | 92 | | 498 | | insulin resistance | | 100 | | 24 | | rosiglitazone (8mg daily) | | Placebo | | 6 months | | 100 | | 3 |
| Tomazic (2005)/ Silic (2007) | Slovenia | 60 | 42.5 | | | 100 | | 348 | | insulin resistance | | 100 | | 100 | | rosiglitazone (4mg daily) | | No Treatment | | 48 weeks | | 100 | | 3 |
| Hadigan (2004) | USA | 28 | 45 | | | 75 | | 469 | | morphologic; insulin resistance | | 100 | | 65 | | rosiglitazone (4mg daily) | | Placebo | | 3 months | | 96 | | 5 |
| Carr (2004)/ Kovacic (2005) | Australia | 108 | 46 | | | 98 | | 576 | | morphologic | | 89 | | 61 | | rosiglitazone (4mg bid) | | Placebo | | 48 weeks | | 95 | | 5 |
| Sutinen (2003)/ Yki-Jarvinen (2003) | Finland | 30 | 43 | | | 83 | | 572 | | morphologic | | 100 | | 80 | | rosiglitazone (8mg daily) | | Placebo | | 24 weeks | | 100 | | 4 |
| **Pioglitazone versus Placebo** | | | | | | | | | | | | | | | | | | | | | | | |  |
| Slama (2006) | France | 130 | 44 | | | 82 | | 590 | | morphologic | | 98 | | 50 | | pioglitazone (30mg daily) | | Placebo | | 48 weeks | | 91 | | 4 |
| Gavrila (2005) | USA | 14 | 48 | | | 93 | | 575 | | insulin resistance; dyslipidemia | | 100 | | 50 | | pioglitazone (30mg daily) | | Placebo | | 1 year | | 100 | | 4 |
| **Metformin versus Placebo or no treatment** | | | |  |  | |  | |  | |  | |  | |  | |  | |  | |  | |  | |
| Kohli (2007) | USA | 48 | 42 | | | 56 | | 394 | | morphologic | | 67 | | 73 | | metformin (1500mg daily) | | Placebo | | 24 weeks | | 81 | | 5 |
| Mulligan (2007) | USA | 53 | 44 | | | 64 | | 609 | | morphologic; insulin resistance | | 95 | | 61 | | metformin (1000mg bid) | | Placebo | | 16 weeks | | 91 | | 4 |
| Tomazic (2005)/ Silic (2007) | Slovenia | 60 | 42.8 | | | 100 | | 340 | | insulin resistance | | 100 | | 100 | | metformin (500mg bid) | | No Treatment | | 48 weeks | | 100 | | 3 |
| Martinez (2003) | Spain | 71 | 41.5 | | | 70 | | NR | | morphologic; dyslipidemia | | 100 | | 100 | | metformin (850mg bid) | | Placebo | | 1 year | | 96 | | 5 |
| Hadigan (2000) | USA | 26 | 45 | | | 76 | | 508 | | morphologic; insulin resistance | | 100 | | 88 | | metformin (500g bid) | | Placebo | | 3 months | | 96 | | 5 |
| St-Marc (1999) | France | 29 | 40 | | | 74 | | 377 | | morphologic; insulin resistance | | NR | | 100 | | metformin (850mg tid) | | No Treatment | | 2 months | | 93 | | 2 |
| **Rosiglitazone vs. Metformin** | |  |  | | |  | |  | |  | |  | |  | |  | |  | |  | |  | |  |
| Mulligan (2007) | USA | 53 | 45 | | | 66 | | 562 | | morphologic; insulin resistance | | 98 | | 71 | | rosiglitazone (4mg daily) | | metformin (1000mg bid) | | 16 weeks | | 89 | | 4 |
| van Wijk (2005)/ Coll (2006) | The Netherlands | 39 | 48 | | | 100 | | 637 | | morphologic | | 100 | | 64 | | rosiglitazone (8mg daily) | | metformin (1000mg bid) | | 6 months | | 95 | | 3 |
| Tomazic (2005)/ Silic (2007) | Slovenia | 60 | 41.8 | | | 100 | | 372 | | insulin resistance | | 100 | | 100 | | rosiglitazone (4mg daily) | | metformin (500mg bid) | | 48 weeks | | 90 | | 3 |
|  |  |  |  | | |  | |  | |  | |  | |  | |  | |  | |  | |  | |  |
| *NRTI=nucleoside reverse transcriptase inhibitor, **PI= protease inhibitor; ***Mulligan study had double placebos; NR=not reported, mg=milligrams, bid=twice a day | | | | | | | | | | | | | | | | | | | | | | | |  |
| ****Jadad Scores range from 0-5, which higher scores indicating better methodologic qualities | | | | | | | | | | | | | | | | | | | | | | | | |
